# Effects of Acute Normobaric Hypoxia on Non-linear Dynamics of Cardiac Autonomic Activity During Constant Workload Cycling Exercise

**DOI:** 10.3389/fphys.2019.00999

**Published:** 2019-08-02

**Authors:** Thomas Gronwald, Olaf Hoos, Kuno Hottenrott

**Affiliations:** ^1^Department of Performance, Neuroscience, Therapy and Health, MSH Medical School Hamburg, Hamburg, Germany; ^2^Center for Sports and Physical Education, Julius Maximilians University of Würzburg, Würzburg, Germany; ^3^Institute of Sports Science, Martin Luther University of Halle-Wittenberg, Halle, Germany

**Keywords:** autonomic nervous system, heart rate variability, detrended fluctuation analysis, endurance exercise, voluntary exhaustion, hypoxia

## Abstract

**Aim:**

Measurements of Non-linear dynamics of heart rate variability (HRV) provide new possibilities to monitor cardiac autonomic activity during exercise under different environmental conditions. Using detrended fluctuation analysis (DFA) technique to assess correlation properties of heart rate (HR) dynamics, the present study examines the influence of normobaric hypoxic conditions (HC) in comparison to normoxic conditions (NC) during a constant workload exercise.

**Materials and Methods:**

Nine well trained cyclists performed a continuous workload exercise on a cycle ergometer with an intensity corresponding to the individual anaerobic threshold until voluntary exhaustion under both NC and HC (15% O_2_). The individual exercise duration was normalized to 10% sections (10–100%). During exercise HR and RR-intervals were continuously-recorded. Besides HRV time-domain measurements (meanRR, SDNN), fractal correlation properties using short-term scaling exponent alpha1 of DFA were calculated. Additionally, blood lactate (La), oxygen saturation of the blood (SpO_2_), and rating of perceived exertion (RPE) were recorded in regular time intervals.

**Results:**

We observed significant changes under NC and HC for all parameters from the beginning to the end of the exercise (10% vs. 100%) except for SpO_2_ and SDNN during NC: increases for HR, La, and RPE in both conditions; decreases for SpO_2_ and SDNN during HC, meanRR and DFA-alpha1 during both conditions. Under HC HR (40–70%), La (10–90%), and RPE (50–90%) were significantly-higher, SpO_2_ (10–100%), meanRR (40–70%), and DFA-alpha1 (20–60%) were significantly-lower than under NC.

**Conclusion:**

Under both conditions, prolonged exercise until voluntary exhaustion provokes a lower total variability combined with a reduction in the amplitude and correlation properties of RR fluctuations which may be attributed to increased organismic demands. Additionally, HC provoked higher demands and loss of correlation properties at an earlier stage during the exercise regime, implying an accelerated alteration of cardiac autonomic regulation.

## Introduction

Over the last 20 years, analytical data on the Non-linear dynamics of a heart rate (HR) time series have been adopted to gain further information of the complex process of cardiovascular regulation Sassi et al. (2015), both at rest and during exercise (Hottenrott and Hoos, 2017; Michael et al., 2017). Thus, measures of complexity of an HR time series, such as heart rate variability (HRV), may aid in monitoring cardiac autonomic activity and in gaining more information on the physiological status of the organismic system during exercise (Aubert et al., 2003). The present state of research suggests that cardiac dynamics is controlled by complex interactions between the two branches of autonomous nervous system, the sympathetic and parasympathetic branch, on the sinus node and other Non-neural factors (Persson, 1996; Gronwald et al., 2019a). These branches compete, resulting in parasympathetic withdrawal and sympathetic activation during exercise (Sandercock and Brodie, 2006). Looking at time- and frequency-domain HRV parameters, even low to moderate exercise intensities induce diminished variability (Gronwald et al., 2019b). During moderate to high exercise intensities, findings from such linear parameters are limited in their informative value and have led to inconsistent results (Hottenrott et al., 2006; Sandercock and Brodie, 2006). Consequently, methods for the Non-linear analysis of HRV were recently developed to detect signal properties that cannot be distinguished by linear analysis techniques (Huikuri et al., 2003; Yeh et al., 2010).

In a healthy state the HRV signal is mainly composed of quasi-periodic oscillations and also possesses fractal structures and random fluctuations (Goldberger et al., 2002). Analyses of these structures have become popular tools and have been shown to be useful diagnostic approaches in the investigation of age and disease (Voss et al., 2009). One widely applied approach to investigate the Non-linear dynamics of HRV and its scaling characteristics is detrended fluctuation analysis (DFA). Originally, this method was developed by Peng et al. (1995) to measure scale-invariant behavior; this involved the evaluation of trends of all sizes in the presence or absence of fractal correlation properties in an HR time series (Yeh et al., 2010). Thus, the DFA method allows to quantify the degree of correlation and fractal scale of an HRV signal resulting in dimensionless measures. The scaling exponents obtained by DFA are also expected to have diagnostic and prognostic abilities, especially for clinical settings. Therefore, the short-term scaling exponent of DFA, called alpha1 (DFA-alpha1), has already been applied for the prognosis of mortality, as well as cardiovascular risk assessment (Peng et al., 1995; Platisa and Gal, 2008; Huikuri et al., 2009; Sen and McGill, 2018).

The current state of research in this field shows that, regardless of the investigated disease or age group, DFA-alpha1 values that differ from the normal value (close to 1.0) (decreasing or increasing) during rest are associated with a higher morbidity or a worse prognosis, revealing a loss of the fractal dynamic toward random (disorganized randomness) or strongly-correlated (periodicity) behavior (de Godoy, 2016). In the context of homeodynamics as well as to system adaptability in response to external (environmental) stressors, that behavior could be interpreted as an effort to maintain basic stability of the control systems between order (persistence) and disorder (change) (Kauffman, 1995; Iyengar et al., 1996; Makikallio et al., 1999; Lipsitz, 2002). Physiologic systems are less adaptable and less able to cope with varied stimuli, such as exposure to different types and modes of exercise or changes in environmental conditions, when they’re losing their fractal complexity (Goldberger, 1997). Hence, physiological complexity reflects the interaction of subsystems and the functioning of organismic regulation as a whole; thus, the higher the complexity, the higher the ability of the system to adapt to different conditions and situations in daily life (Goldberger et al., 2002; Gronwald et al., 2019a).

The related explanations on the Non-linear dynamics and complexity of organismic stability and self-regulation also seem to apply in endurance sports. In this respect, approaches and models from sports medicine and exercise science refer, on the one hand, to the importance of the brain in the control of fatigue processes and endurance performance and, on the other hand, to the complexity of the control and regulation processes of the subsystems limiting endurance performance (Noakes et al., 2004; Abbiss and Laursen, 2005; Marcora, 2008; Ament and Verkerke, 2009; Millet, 2011; Noakes, 2011, 2012; St Clair Gibson et al., 2018). HRV, as a marker of the integrated response of the heart to the complex, Non-linear interaction of sympatho-vagal activity and other factors could provide an adequate methodological approach, as it results from a complex central-peripheral integration of information from different cardiovascular feedback mechanisms and the central command within the central autonomic network (CAN) (Benarroch, 1993; Williamson et al., 2006).

As DFA, and its short-term scaling exponent alpha1, has a low dependence on HR and provides robustness against artifacts (Peng et al., 1995; Sandercock and Brodie, 2006; Silva et al., 2017), this method seems to be suitable for analyzing the complexity of cardiovascular regulation during endurance exercise with various exercise modalities and intensities (Gronwald et al., 2019a). Through the easy detection of HRV with a chest strap, DFA-alpha1 could be also useful as a diagnostic or monitoring metric for endurance trained athletes for assessing dose-response relationship in combination with other applicable internal and external load parameters.

Consequently, some studies have used DFA to analyze a time series during different types and modes of exercise (Tulppo et al., 2001; Hautala et al., 2003; Casties et al., 2006; Platisa et al., 2008; Hottenrott and Hoos, 2017; Gronwald et al., 2018, 2019a,b). However, further studies are necessary to analyze different modes of exercise, as well as changing environmental factors, such as hypoxic conditions (HC) or heat and cold exposure, to gain new insights for the suitability of DFA-alpha1 as a control or monitoring parameter in endurance exercise training (Gronwald et al., 2019b).

A recent systematic review by Oliveira et al. (2017) showed that acute exposure to hypoxia under resting conditions substantially-changes the HRV of healthy individuals (both time- and frequency-domain), and results in a decrease in the cardiac autonomic modulation. This is proposed to occur by either reducing or maintaining vagal modulation, by enhancing sympathetic activation, or even by a combination of these responses. The described responses are mainly dependent on the altitude level, length of exposure, interindividual variation, and barometric pressure in the comparison of the effects of normobaric versus hypobaric hypoxia. Until now, only a few studies have analyzed the influence of hypoxia on the Non-linear parameters of HRV in general, and specifically DFA. Neither Zhang et al. (2015) or Vigo et al. (2010) could detect an effect of hypobaric hypoxia (up to 3600 and 8230 m, respectively) on DFA-alpha1 while sitting. Although there was no significant decrease detected during exposure to high hypobaric hypoxia by Vigo et al. (2010), Giles et al. (2016) found a significant increase in DFA-alpha1 during exposure to normobaric hypoxia up to 6000 m (9.8% O_2_) during supine recordings. To the best of our knowledge, there are no studies to date that have analyzed the influence of hypoxia on the correlation properties of HRV (detected by DFA and its short-term scaling exponent alpha1) during exercise.

Therefore, the aim of the present study was to evaluate differences in the influence of a continuous workload exercise bout under both normoxic and normobaric HCs in terms of standard time-domain measures and Non-linear dynamics of HRV. The main objective was to determine whether characteristics of the short-term scaling exponent of DFA toward a random signal under acute prolonged exercise until voluntary exhaustion may differ in response to HCs. The ultimate goal of this study was to gain further insight into the complex organismic regulation and fatigue dynamics during exercise.

## Materials and Methods

### Participants

Nine endurance trained male cyclists were recruited from local sports clubs. We included Non-smoking adults who performed cycling training sessions for at least 12 h per week (300–450 km/week) in the 6 months before the start of the study but no altitude training or visits above 1000 m in the 3 months before the start of the study. As stated in the investigation of Gronwald et al. (2019a) a preliminary medical check-up, following the S1 guidelines of the German Association for Sports Medicine and Prevention, was performed to ensure that the participants were free from cardiovascular, neurologic, pulmonary, and orthopedic problems. The check-up also included an ECG at rest and a personal anamnesis. This study protocol was approved by the ethics committee of the University Clinic of Halle (Saale), Medical Faculty of Martin Luther University of Halle-Wittenberg and was performed in accordance with the guidelines of the Helsinki World Medical Association Declaration. Following explanation of any risks and benefits associated with the study, all participants approved the study and written informed consents were obtained.

### Procedure

**FIGURE 1:**
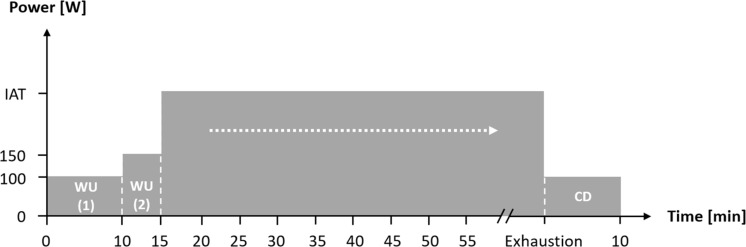
Flow chart of the constant workload until voluntary exhaustion during normoxic and normobaric hypoxic condition. IAT: individual anaerobic threshold; WU (1): Warm-Up at 100 W; WU (2): Warm-Up at 150 W; CD: Cool-Down at 100 W.

First, aerobic fitness was assessed using spiroergometry (Metamax 3b, Cortex, Germany) during an incremental test until the point of voluntary exhaustion (start: 100 W, increment: 20 W, length: 3 min, cadence: 80–90 rpm) on a high-performance bicycle ergometer (E 2000 s, FES, Germany). On the basis of the study by Dickhuth et al. (1999), the individual anaerobic threshold (IAT) was derived from the lactate-power curves. One and two weeks after the first laboratory visit, participants completed an exercise bout of continuous workload under both normoxic (NC) and normobaric HCs, with the same exercise intensity at IAT until voluntary exhaustion (Figure 1). The test conditions were randomized, single-blinded, and performed in a laboratory hypoxic chamber on the same bicycle ergometer and at the same daytime. Participants were instructed and encouraged to keep a nutrition intake and training load diary in order to keep influences at a minimum. Before the exercise bout, there was a warm-up period at 100 W for 10 min, followed by 150 W for 5 min. After the exercise bout there was a cool-down period at 100 W over a period of 10 min (see Figure 1).

### Normobaric Hypoxic Conditions

A hypoxic chamber was used (Höhenbalance AG, Germany) for the supply of oxygen deficiency conditions (hypoxia, low oxygen concentration) at constant pressure conditions (normobaric) in the breathing air. The chamber had four b-Cat high-altitude-generators with an air conditioning system. To generate oxygen deficiency conditions, the four generators exchanged oxygen proportional to nitrogen (change in the O_2_-N_2_-ratio). The oxygen and carbon dioxide concentration in the breathing air were constantly measured and checked using a sensor system. In addition, the carbon dioxide content was maintained under a level of 0.3% via a ventilation system. The room temperature (20°C) and humidity (50%) were controlled by air conditioning of the chamber. The condition in the hypoxic chamber corresponded to normal laboratory conditions. The exercise bout under normobaric HCs was performed at a percentage of oxygen (oxygen fraction: FiO_2_) in the breathing air of 15.0%, corresponding to an increase in simulated height of approximately 2700 m. The test under normoxic (sea-level) conditions (FiO_2__,_ 20.9%) was performed in the same chamber.

### Measurements

During exercise, the HR and RR-intervals were recorded continuously (beat-to-beat-modus) using a HR-monitor with a time resolution of 1 ms (Polar s810i, Polar Electro GmbH, Germany; Weippert et al., 2010). Collected raw data were transferred to a PC via an infrared interface, and artifacts were subsequently detected with a semi-automatic approach. The RR-intervals, which were distinguished by more than a 30% difference from the previous interval, were determined as artifacts. Artifacts were replaced with the average calculated from the previous and subsequent values, and data sets with more than 5% of artifacts were completely excluded from further processing. Only the NN intervals (normal-to-normal intervals) were considered during the data analysis (Task Force, 1996). The NN intervals were stored as ASCII files for further data analysis.

Using Kubios HRV software (Version 2.1, Biosignal Analysis and Medical Imaging Group, Kuopio, Finland; Tarvainen et al., 2014), the HRV analysis was conducted on data collected from the last 2 min of the warm-up and cool-down periods. In addition, during prolonged exercise segments of 2 min were analyzed every 5 min and before voluntary exhaustion. Besides the standard parameters obtained from time-domain analysis, including the average of the normal RR-interval length (meanRR in ms) and the total variability as the standard deviation of all normal RR-intervals (SDNN in ms), the scaling behavior was calculated using the Non-linear short-term scaling exponent DFA-alpha1. DFA has been referred to as a modification of the root mean square analysis (RMS) that is also suitable for analyzing short and Non-stationary time series data (Peng et al., 1995). Briefly, the RMS fluctuation of the integrated and detrended data is measured in observation windows of different sizes; the data are then plotted against the size of the window on a log–log scale. The scaling exponent represents the slope of the line, which relates the (log) fluctuation to the (log) window size (Mendonca et al., 2010). In this study, we only computed the short-term scaling exponent (window width: 4 ≤ *n* ≤ 16 beats) because of the relatively short recording times for each condition (Tulppo et al., 2001; Hautala et al., 2003). The DFA-alpha1 values indicate time series fractal correlation properties, such as the type of noise (approximately 1.5 for strongly correlated Brownian noise and ≤ 0.5 for uncorrelated white noise with random signals). A value of approximately 1 signifies a mix of uncorrelated and maximally correlated signal components with 1/*f* noise; this represents a balance between the complete unpredictability (randomness) of white noise and the predictability (strong correlation) of Brownian noise (Platisa and Gal, 2008). The exponent is also an indicator of the “roughness” of the time series, with larger values of DFA-alpha1 representing a smoother time series (Peng et al., 1995; Goldberger et al., 2002).

Additionally, the blood lactate (La) and blood glucose (Glu) concentrations were assessed with Super GL ambulance (Dr. Mueller, Germany) from blood taken from an earlobe (Faude and Meyer, 2008). The oxygen saturation of the blood (SpO_2_) was assessed via pulsoximetry (PM-60 OxiFlex, Mindray, Germany), and participants were asked to rate their perceived exertion (RPE: 6–20; Borg, 1982). These measures were taken every 5 min of the constant workload and after voluntary exhaustion. All parameters were also assessed during the end of the warm-up period, the end of the cool-down period, and in the resting state (with the exception of RPE).

### Statistical Analysis

The statistical analysis was performed using SPSS 23.0 (IBM Statistics, United States) for Windows (Microsoft, United States). The Shapiro–Wilk test was applied to verify the Gaussian distribution of the data. The degree of variance homogeneity was verified by the Levene test. To analyze the effects of the exercise bout on dependent variables (HR, HRV parameters, La, SpO_2_, and RPE) under the two conditions (NC, HC), a two-way ANOVA (factors: condition, time), with repeated measures, was applied. The main effects and interaction (condition × time) were reported and *post hoc* tests (Bonferroni) were applied to compare the differences between conditions. For the comparison of the dependent variables in the warm-up period, the cool-down period, and between the two conditions, the paired *t*-test was used. For all tests, the statistical significance was accepted as *p* ≤ 0.05. η^2^ was used to denote the effect sizes of main effects (small effect = 0.01, medium effect = 0.06, large effect = 0.14; Döring and Bortz, 2016) and Cohen’s *d* for effect sizes in comparison of the measurement intervals (small effect = 0.2, medium effect = 0.5, large effect = 0.8; Cohen, 1988).

Because of the performance heterogeneity of the participants, the measures recorded during the constant workload were not statistically tested with the absolute test duration as an independent variable. To standardize and improve the comparability, the recorded data were normalized in relation to the individual total test duration. Therefore, before statistical processing of the data, all measures of all participants were interpolated to 10% steps using the cubic spline algorithm on MS Excel (Microsoft, United States). The preload value was defined as 0% in each case (Warm-Up at 150 W). The equidistant percentage segments calculated in this way enabled multiple statistical comparisons.

## Results

Participants (age: 26.4 ± 4.1 years; height: 181.7 ± 5.3 cm; body mass: 79.2 ± 9.3 kg; body fat: 13.2 ± 3.6%; VO_2_peak: 53.1 ± 4.7 ml/min/kg) achieved a maximum power output of 342.2 ± 28.3W during the incremental cycling exercise test. The constant cycling bout under normoxic and normobaric HCs was performed at 266.2 ± 26.3 W (IAT) which corresponds to 80.8 ± 9.4% of peak oxygen uptake (VO_2_peak) in the incremental test. Compared to the HC, participants obtained a significant longer duration during the normoxic condition (NC) until voluntary exhaustion (NC: 41:18 ± 08:21 min:sec vs. 24:42 ± 06:09min:sec; *p* < 0.001, *d* = 1.553). Maximum RPE values of 19.7 ± 0.7 (NC) vs. 19.6 ± 0.7 (HC) indicate a voluntary exhaustion during both conditions (*p* = 0.738, *d* = 0.053).

A significant main effect for condition could be found for La, SpO_2_, RPE and DFA-alpha1. Despite for SpO_2_, for all analyzed parameters a significant main effect of time could be determined. In addition, a significant main effect of interaction (condition × time) could be found for HR, La, RPE and meanRR, while SDNN and DFA-alpha1 showed a statistical trend. Detailed ANOVA results and descriptive values of all analyzed parameters during rest and over the time course of exercise during NC and HC are provided in Table 1.

**TABLE 1:** Heart rate, oxygen saturation, lactate, rating of perceived exertion and HRV measures (Mean ± SD) during resting state and all cycling conditions during normoxia (NC) and normobaric hypoxia (HC).

**Parameters**	**Condition**	**Rest**	**WU (1)**	**WU (2)**	**10%**	**20%**	**30%**	**40%**	**50%**	**60%**	**70%**	**80%**	**90%**	**100%**	**CD**
HR [1/min]	NC	72.2 ± 5.7	108,4^*^ ± 10.1	120,5^*^ ± 13.6	147,2^*^ ± 11.5	155,0^*^ ± 10.9	157,3^*^ ± 11.0	159,1 ± 10.3	160,3 ± 9.6	161,4 ± 9.3	163,9^*^ ± 9.4	169,0^*^ ± 8.5	174,7^*^ ± 7.9	180,5^*^¥± 9.0	126,9^*^§ ± 11.0
	HC	69.3 ± 8.8	111.8^*^ ± 10.0	127.2^*^ ± 12.0	145.9^*^ ± 9.9	159.3^*^ ± 8.7	165.3^*^ ± 7.0	167.6# ± 6.4	169.1# ± 6.3	170.6# ± 6.2	172.3^*^# ± 6.0	173.6^*^ ± 6.0	175.1^*^ ± 6.6	176.8¥± 7.6	127.3^*^§ ± 12.0

ANOVA	10–100% – Condition: F = 3.348, *p* = 0.105, η^2^ = 0.295; Time: F = 181.496, *p* < 0.001, η^2^ = 0.958; Interaction: F = 15.638, *p* < 0.001, η^2^ = 0.662														

SpO_2_ [%]	NC	98.0 ± 0.5	97.1^*^ ± 0.8	97.1 ± 0.6	96.0 ± 1.1	95.8 ± 1.3	96.1 ± 1.0	96.0 ± 0.7	95.7 ± 0.8	95.8 ± 0.8	95.9 ± 0.8	95.9 ± 0.8	95.5 ± 1.1	95.9 ± 1.1	97.7^*^§ ± 0.5
	HC	92.4# ± 2.7	86.6^*^# ± 3.9	85.2# ± 2.7	84.5# ± 2.7	84.0# ± 2.8	83.7# ± 2.7	83.4# ± 2.3	83.3# ± 2.1	83.3# ± 2.1	83.4# ± 2.3	83.4# ± 2.3	83.1# ± 2.1	82.7#¥± 2.2	91.1^*^§ # ± 2.5

ANOVA	10–100% – Condition: *F* = 591.301, *p* < 0.001, η^2^ = 0.987; Time: *F* = 2.266, *p* = 0.121, η^2^ = 0.221; Interaction: *F* = 1.901, *p* = 0.163, η^2^ = 0.192														

La [mmol/l]	NC	1.03 ± 0.27	0.72^*^ ± 0.30	0.94 ± 0.62	2.19^*^ ± 0.92	2.65 ± 1.28	2.70 ± 1.63	2.67 ± 1.90	2.69 ± 2.07	2.78 ± 2.27	3.11 ± 2.41	3.72 ± 2.53	4.75 ± 2.44	6.89¥± 2.09	3.30^*^§ ± 1.59
	HC	0.73 ± 0.21	0.65 ± 0.15	0.86^*^ ± 0.33	2.94^*^# ± 0.39	4.55^*^# ± 0.58	5.48^*^# ± 0.79	5.98^*^# ± 0.94	6.31# ± 1.26	6.68# ± 1.73	7.13# ± 2.06	7.49# ± 2.09	7.76# ± 1.97	8.14¥± 1.78	3.61^*^§ ± 0.96

ANOVA	10–100% – Condition: *F* = 31.402, *p* = 0.001, η^2^ = 0.797; Time: *F* = 40.337, *p* < 0.001, η^2^ = 0.834; Interaction: *F* = 9.395, *p* = 0.001, η^2^ = 0.540														

RPE [6-20]	NC	–	7.2 ± 1.6	8.8^*^ ± 1.9	13.0^*^ ± 1.3	14.1^*^ ± 1.0	14.2 ± 1.2	14.5 ± 1.2	15.0 ± 1.1	15.1 ± 1.4	15.5 ± 1.5	16.5 ± 1.2	17.3 ± 1.5	19.7¥± 0.7	7.9^*^ ± 2.0
	HC	–	7.2 ± 1.3	9.4^*^ ± 1.8	12.4^*^ ± 1.4	14.4^*^ ± 1.2	15.2 ± 1.3	15.8 ± 1.3	16.3# ± 1.1	16.8# ± 1.2	17.5# ± 1.4	18.1# ± 1.4	18.7# ± 1.1	19.6¥± 0.7	7.9^*^ ± 1.5

ANOVA	10–100% – Condition: *F* = 45.320, *p* < 0.001, η^2^ = 0.850; Time: *F* = 99.726, *p* < 0.001, η^2^ = 0.926; Interaction: F = 6.840, *p* = 0.006, η^2^ = 0.461														

meanRR [ms]	NC	842 ± 66	558^*^ ± 49	503^*^ ± 52	411^*^ ± 34	388^*^ ± 28	383 ± 28	378 ± 25	376 ± 22	373 ± 21	367^*^ ± 21	356^*^ ± 18	344^*^ ± 16	333^*^¥± 17	476^*^§ ± 41
	HC	884 ± 99	538^*^ ± 53	471^*^ ± 48	417^*^ ± 32	379^*^ ± 22	362^*^ ± 15	358# ± 14	356# ± 13	352# ± 13	349^*^# ± 12	346^*^ ± 12	343^*^ ± 13	340¥± 15	474^*^§ ± 46

ANOVA	10–100% – Condition: *F* = 2.931, *p* = 0.125, η^2^ = 0.268; Time: F = 110.715, *p* < 0.001, η^2^ = 0.933; Interaction: F = 10.399, *p* = 0.001, η^2^ = 0.565														

SDNN [ms]	NC	66.5 ± 17.5	8.7^*^ ± 3.0	5.7^*^ ± 1.9	2.6^*^ ± 0.7	2.1 ± 0.4	2.3 ± 0.3	2.1 ± 0.3	2.0 ± 0.3	2.0 ± 0.3	1.9 ± 0.4	1.9 ± 0.4	2.0 ± 0.5	2.1 ± 0.5	3.6^*^§ ± 1.2
	HC	82.3 ± 21.9	6.8^*^ ± 1.8	4.2^*^ ± 1.5	2.9 ± 0.7	2.1 ± 0.5	1.9 ± 0.4	1.9 ± 0.4	2.0 ± 0.5	2.1 ± 0.5	2.1 ± 0.4	2.1 ± 0.5	2.2 ± 0.6	2.3¥± 0.6	3.3^*^§ ± 0.7

ANOVA	10–100% – Condition: F = 0.340, *p* = 0.576, η^2^ = 0.041; Time: F = 8.560, *p* = 0.001, η^2^ = 0.517; Interaction: F = 2.641, *p* = 0.062, η^2^ = 0.248														

DFA-alpha1 []	NC	1.35 ± 0.13	1.49^*^ ± 0.16	1.27^*^ ± 0.25	0.79^*^ ± 0.14	0.66 ± 0.14	0.62 ± 0.18	0.54 ± 0.16	0.50 ± 0.13	0.51 ± 0.11	0.47 ± 0.12	0.38 ± 0.12	0.31 ± 0.14	0.31¥± 0.11	1.18^*^§ ± 0.28
	HC	1.26 ± 0.10	1.44^*^ ± 0.18	1.11^*^ ± 0.25	0.77^*^ ± 0.16	0.53^*^# ± 0.13	0.41# ± 0.08	0.38# ± 0.08	0.39# ± 0.12	0.36# ± 0.14	0.34 ± 0.18	0.32 ± 0.16	0.32 ± 0.15	0.33¥± 0.12	0.99^*^§ ± 0.20

ANOVA	10–100% – Condition: *F* = 26.098, *p* = 0.001, η^2^ = 0.765; Time: *F* = 49.257, *p* < 0.001, η^2^ = 0.860; Interaction: *F* = 2.897, *p* = 0.080, η^2^ = 0.266														

WU (1): Warm-Up at 100 W; WU (2): Warm-Up at 150 W; 10–100%: Percentage of continuous workload; CD: Cool-Down at 100 W. Main effects (factors: condition, time) and interaction (condition × time) of the two-way ANOVA with repeated measures. HR: heart rate, SpO_2_: oxygen saturation of the blood, La: blood lactate concentration, RPE: rate of perceived exertion, meanRR: average of normal RR-intervals, SDNN: standard deviation of all normal RR-intervals, DFA-alpha1: short-term scaling exponent of detrended fluctuation analysis; ^*^Significant compared to preceding measurement; §Significant change WU (1) vs. CD at 100W; ¥Significant change 10% vs. 100%; #Significant change NC vs. HC (*p* ≤ 0.05).

**FIGURE 2:**
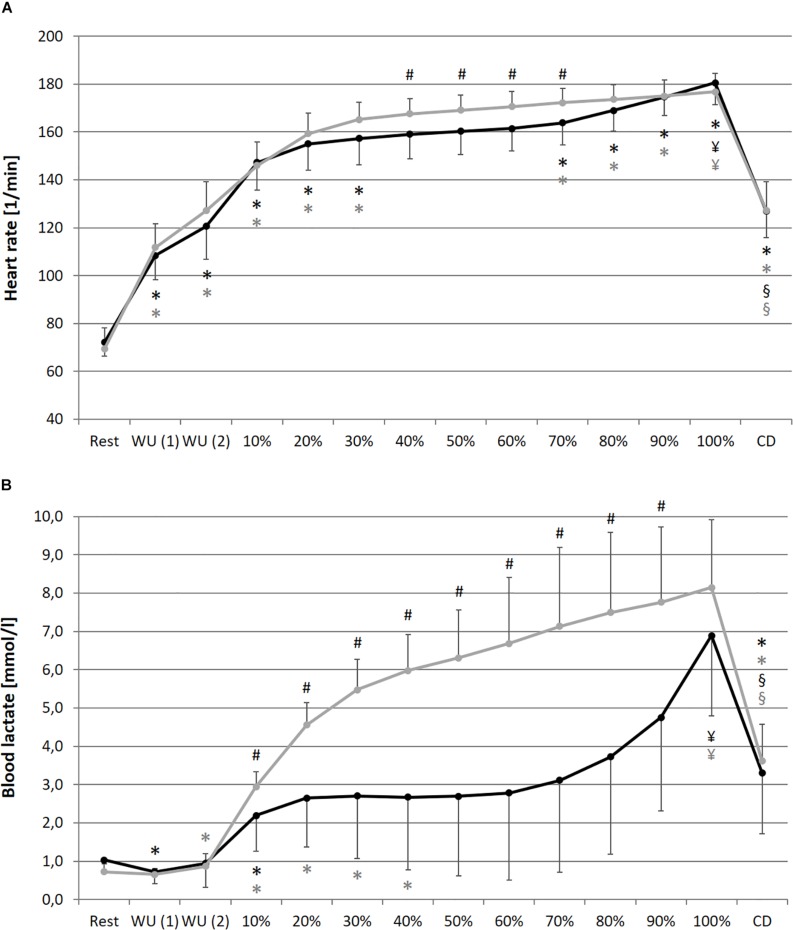
Heart rate **(A)**, blood lactate concentration **(B)** during resting state and all cycling conditions during normoxia (NC, black color) and normobaric hypoxia (HC, gray color). WU (1): Warm-Up at 100W; WU (2): Warm-Up at 150W; 10–100%: Percentage of continuous workload; CD: Cool-Down at 100W. ^*^Significant compared to preceding measurement; §Significant change WU (1) vs. CD at 100W; ¥Significant change 10% vs. 100%; #Significant change NC vs. HC (*p* ≤ 0.05).

**FIGURE 3:**
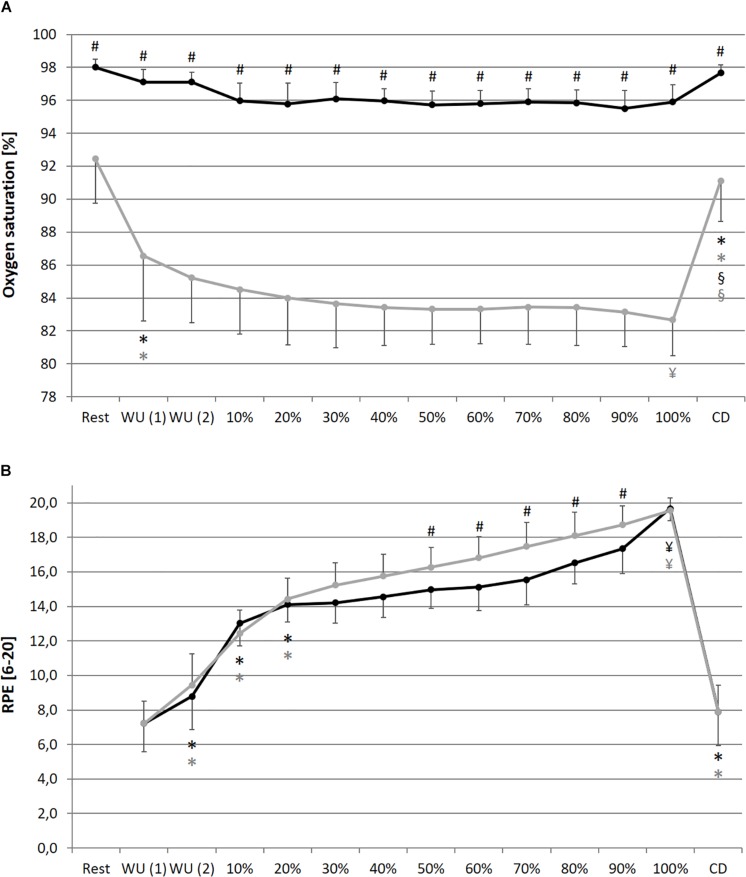
Oxygen saturation of the blood **(A)** and rate of perceived exertion RPE, **(B)** during resting state and all cycling conditions during normoxia (NC, black color) and normobaric hypoxia (HC, gray color). WU (1): Warm-Up at 100 W; WU (2): Warm-Up at 150 W; 10–100%: Percentage of continuous workload; CD: Cool-Down at 100 W. ^*^Significant compared to preceding measurement; §Significant change WU (1) vs. CD at 100 W; ¥Significant change 10% vs. 100%; #Significant change NC vs. HC (*p* ≤ 0.05).

**FIGURE 4:**
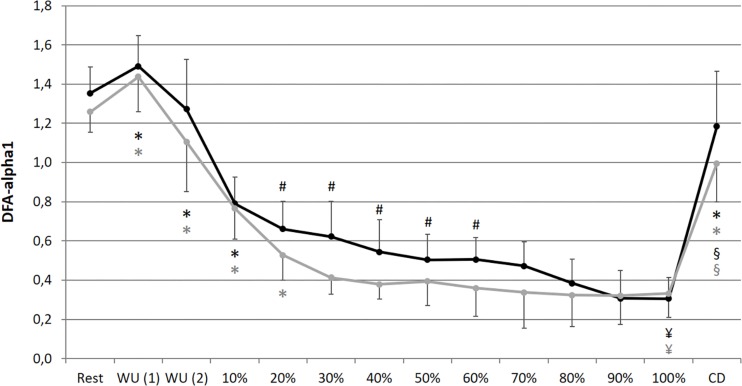
Short-term scaling exponent (DFA-alpha1) during resting state and all cycling conditions during normoxia (NC, black color) and normobaric hypoxia (HC, gray color). WU (1): Warm-Up at 100 W; WU (2): Warm-Up at 150 W; 10–100%: Percentage of continuous workload; CD: Cool-Down at 100 W. ^*^Significant compared to preceding measurement; §Significant change WU (1) vs. CD at 100 W; ¥Significant change 10% vs. 100%; #Significant change NC vs. HC (*p* ≤ 0.05).

In comparison of the beginning and end of the prolonged exercise bout (10% vs. 100%) during NC and HC significant changes could be found in all measures except for SpO_2_ and SDNN during NC; increases for HR, La and RPE during both conditions; decreases for SpO_2_ and SDNN during HC, meanRR and DFA-alpha1 during both conditions (NC – HR: *p* < 0.001, *d* = 2.921; La: *p* < 0.001, *d* = 2.621; SpO_2_: *p* = 0.652, *d* = 0.074; RPE: *p* < 0.001, *d* = 5.220; meanRR: *p* < 0.001, *d* = 1.766; SDNN: *p* = 0.081, *d* = 0.762; DFA-alpha1: *p* < 0.001, *d* = 3.943; HC – HR: *p* < 0.001, *d* = 3.322; La: *p* < 0.001, *d* = 2.561; SpO_2_: *p* = 0.016, *d* = 0.728; RPE: *p* < 0.001, *d* = 6.388; meanRR: *p* < 0.001, *d* = 2.368; SDNN: *p* = 0.035, *d* = 0.973; DFA-alpha1: *p* < 0.001, *d* = 3.097). In summary, we found a decrease in total variability combined with a reduction in the amplitude and correlation properties of RR fluctuations during prolonged exercise. In comparison of the first warm-up period and the cool-down period (WU (1) vs. CD) both of 10 min duration and at 100 W, significant changes could be found in all measures except for RPE; increases for HR, SpO_2_ and La; decreases for meanRR, SDNN and DFA-alpha1 (NC – HR: *p* < 0.001, *d* = 1.692; La: *p* = 0.001, *d* = 2.073; SpO_2_: *p* = 0.013, *d* = 0.751; RPE: *p* = 0.347, *d* = 0.366; meanRR: *p* < 0.001, *d* = 1.588; SDNN: *p* < 0.001, *d* = 1.754; DFA-alpha1: *p* = 0.020, *d* = 1.337; HC – HR: *p* < 0.001, *d* = 1.374; La: *p* < 0.001, *d* = 4.446; SpO_2_: *p* = 0.024, *d* = 1.393; RPE: *p* = 0.141, *d* = 0.463; meanRR: *p* = 0.001, *d* = 1.260; SDNN: *p* < 0.001, *d* = 2.544; DFA-alpha1: *p* = 0.001, *d* = 2.359) (see Figures 2–4).

In comparison of the condition, we found significant higher values during prolonged exercise and HC of HR at 40–70% (40%: *p* = 0.050, *d* = 0.653; 50%: *p* = 0.036, *d* = 0.659; 60%: *p* = 0.025, *d* = 0.707; 70%: *p* = 0.037, *d* = 0.676), of La at 10–90% (10%: *p* = 0.038, *d* = 0.766; 20%: *p* = 0.001, *d* = 1.403; 30%: *p* < 0.001, *d* = 1.450; 40%: *p* < 0.001, *d* = 1.331; 50%: *p* < 0.001, *d* = 1.263; 60%: *p* = 0.001, *d* = 1.371; 70%: *p* = 0.002, *d* = 1.293; 80%: *p* = 0.003, *d* = 1.098; 90%: *p* = 0.011, *d* = 1.009) and of RPE at 50–90% (50%: *p* = 0.025, *d* = 0.625; 60%: *p* = 0.013, *d* = 0.846; 70%: *p* = 0.011, *d* = 0.885; 80%: *p* = 0.019, *d* = 0.611; 90%: *p* = 0.036, *d* = 0.659). Significant lower values during prolonged exercise and HC could be shown for SpO_2_ at 10–100% (10%: *p* < 0.001, *d* = 2.541; 20%: *p* < 0.001, *d* = 2.613; 30%: *p* < 0.001, *d* = 3.775; 40%: *p* < 0.001, *d* = 3.002; 50%: *p* < 0.001, *d* = 3.315; 60%: *p* < 0.001, *d* = 3.597; 70%: *p* < 0.001, *d* = 3.852; 80%: *p* < 0.001, *d* = 3.399; 90%: *p* < 0.001, *d* = 4.636; 100%: *p* < 0.001, *d* = 4.699), for meanRR at 40–70% (40%: *p* = 0.047, *d* = 0.675; 50%: *p* = 0.035, *d* = 0.659; 60%: *p* = 0.022, *d* = 0.713; 70%: *p* = 0.035, *d* = 0.687), and for DFA-alpha1 at 20–60% (20%: *p* = 0.050, *d* = 0.691; 30%: *p* = 0.006, *d* = 1.216; 40%: *p* = 0.014, *d* = 0.739; 50%: *p* = 0.050, *d* = 0.428; 60%: *p* = 0.028, *d* = 0.604) (see Figures 2**–**4).

## Discussion

The presented data demonstrate that prolonged exercise until voluntary exhaustion provokes a lower total variability combined with a reduction in the amplitude and correlation properties of RR fluctuations. This may be attributed to increased organismic demands under NC and HC and could be confirmed by other parameters, such as increases in HR, La, and RPE and decreases in SpO_2_. In addition, our data implies that HC provoked higher demands and loss of correlation properties at an earlier stage during the exercise regime compared to normoxia, implying an accelerated alteration of cardiac autonomic regulation. DFA-alpha1 was most sensitive to this difference by showing a large main effect for condition (η^2^ of 0.765) and discriminating even at early relative exercise durations of 20%, while HR and meanRR failed to do so until 40–70% of the prolonged exercise. In this regard, DFA-alpha1 could provide added value in the interpretation of the hypoxic effects during exercise. This observation is in line with the results of previous studies supporting higher values of HR, La, and RPE and considerably lower values of SpO_2_ during exercise under hypoxic compared with NCs (Bärtsch and Gibbs, 2007; Ofner et al., 2014; Moon et al., 2016; Deb et al., 2018). However, the magnitude of cardiopulmonary responses to a certain intensity of hypoxia and exercise is intra-individual (Deb et al., 2018; Wehrlin and Hallén, 2006). These differences could be interpreted as an acute compensation response to reduced aerobic exercise availability by decreased oxygen delivery and utilization capacities under HC. The higher demands result in a shorter exercise duration until voluntary exhaustion during HC. It should be noted that, through the maintenance of the required power output for as long as possible and the absence of a known endpoint, the exercise regime could be classified as “not self-paced” and “open-loop” (Smirmaul et al., 2013). Open-loop exercises require a simple behavioral decision of “continue” or “stop” by the participants. During self-regulated or self-paced (closed-loop) exercise, participants are able to compensate by voluntarily changing the power or speed at which they are performing the task, through pacing. With reference to the earlier loss of correlation properties due to DFA-alpha1 during HC (especially during the first half of the prolonged exercise), to date there is only little information on the influence of different kinds of hypoxia on DFA-alpha1 during resting conditions (Vigo et al., 2010; Zhang et al., 2015; Giles et al., 2016), but not during exercise.

However, the decrease in DFA-alpha1 may verify a demand-dependent change from strongly correlated behavior in the warm-up periods, to uncorrelated/stochastic or anti-correlated behavior of the RR-intervals during prolonged exercise in both conditions (Platisa and Gal, 2008). This is consistent with previous studies reporting an almost linear reduction in complexity and correlation properties with a gradual change of the RR data structure towards an anti-correlated and merely random signal for medium-to-high exercise intensity demands (Karasik et al., 2002; Hautala et al., 2003; Casties et al., 2006; Platisa et al., 2008; Hottenrott and Hoos, 2017; Blasco-Lafarga et al., 2017; Gronwald et al., 2018, 2019a,b). Due to increased sympathetic activity and/or decreased parasympathetic activity during endurance exercise, the loss of complexity could be related to the disruption of the equilibrium and interaction between the two branches of the autonomic nervous system (Sandercock and Brodie, 2006; Lewis and Short, 2010). This particular change could be due to a protection of homeodynamic processes through an organismic system withdrawal (Casties et al., 2006; Platisa et al., 2008), which are matched by the CAN, integrating various internal and external stimuli (Benarroch, 1993, 1997). The great loss of complexity might also be a consequence of complementary neural mechanisms/circuits (Shaffer et al., 2014) which aim the maintenance of locomotor-respiratory coupling in the context of coordination between movement frequency (e.g., cadence in cycling exercise), heartbeat and breathing patterns during cycling exercise (Casties et al., 2006; Blasco-Lafarga et al., 2017; Gronwald et al., 2018, 2019a).

A possible explanatory approach for the decrease in HRV complexity during endurance exercise could be an increased reduction of the input number of different physiological systems, and/or the reduction of the interaction of various subsystems with a particular focus on either one dominant system or a few dominant systems (Nakamura et al., 1993; Casties et al., 2006). This could be interpreted in the sense of centralization or “mechanization” of a complex physiological system (von Bertalanffy, 1950). In the sense of this mechanization of the organism regulation, a dominant “performance attractor” could emerge during high physiological demand, which could be determined by sympathetic activity (Karasik et al., 2002; Hautala et al., 2003), neuro-mechanical coupling of several oscillators (Casties et al., 2006), and/or Non-neural, intrinsic HR regulation (Platisa and Gal, 2008). Thus, every fluctuation is corrected immediately in the opposite direction by the dominant attractor, which results in a random or anti-correlated signal. This organismic system withdrawal may also be interpreted as a loss of systemic integrity, in the sense of a hazardous situation for homeostasis (Seely and Macklem, 2004).

In this regard, a link between the complexity measures of HRV and their connectivity from an autonomic nervous system point of view might offer new perspectives to evaluate complex models of exercise fatigue and endurance performance. The field of research contains numerous models that emphasize the importance of the brain for the control and regulation of organismic processes during endurance exercise (Abbiss and Laursen, 2005; Marcora, 2008; Ament and Verkerke, 2009; Millet, 2011: Noakes, 2011, 2012; St Clair Gibson et al., 2018) and other concepts of cardiovascular control during exercise that focus on central command (Boulpaep, 2009; Williamson, 2010). Some of the models have been much debated in the past (e.g., Amann and Secher, 2010; Marcora, 2010; Perrey et al., 2010). However, it should be noted that in this debate, Smirmaul et al. (2010) puts the discussion in a nutshell, so that the role of central control cannot be ignored (Noakes, 2011), the cognitive and motivational factors proposed by Marcora (2010) comprise key components of exercise tolerance and endurance performance; afferent feedback from motor muscles (Amann and Secher, 2010) is also important (but not the sole factor directly limiting endurance exercise performance) since it acts to increase the conscious awareness of organismic “discomforts.” This holistic approach, with the complex interaction of all of these factors, allows for more appropriate behavioral decisions and is crucial, especially for endurance exercise performance. Finally, fatigue processes are complex, and their analysis and understanding should not be reduced to a single isolated factor.

In this context, the Non-linear dynamics of HRV might provide a new explanatory approach. The integration of peripheral and central information for the self-controlled down regulation and limitation of muscle recruitment as a protection mechanism for organismic homeostasis, as hypothetized in the “central governor model” (Noakes, 2011, 2012), could be described in more detail as follows (Gronwald et al., 2018). On the basis of the interaction between cardiovascular feedback mechanisms and the central command within the CAN (Benarroch, 1993), this could lead to an accumulation of afferent feedback disturbances modified by many physiological systems (e.g., relevant sensory information, previous sport specific experiences, load anticipation, and current environmental conditions). This results in a multi-feedback system which regulates physiologic processes during exercise (Gronwald et al., 2018).

The study group of Noakes et al. (2001) also provides evidence that peak cardiovascular function and peak skeletal muscle electromyographic activity is reduced during maximal exercise in hypoxia. The authors concluded that the presented data support a model in which a central neural governor constrains the cardiac output by regulating the mass of skeletal muscle that can be activated during maximal exercise. Although there is no difference between NC and HC in the presented data during voluntary exhaustion for all analyzed measures, with the exception of SpO_2_, this indicates voluntary exhaustion during both conditions, with maximum values of RPE. However, if we take a closer look at the HR data, we can determine slightly (not significant) decreased maximum values during HC compared to NC. Additionally, there was only a further increase in HR data from 90 to 100% during NC, and a leveling off during HC. These observations could support the concluding remarks of Noakes et al. (2001).

In support of such a model, parameters such as blood lactate concentration (here with a substantially-accelerated increase during HC) can be considered to be indicative of functions of a signal molecule for peripheral-central information exchange (Philp et al., 2005), which, in conjunction with other psycho-physiological variables (e.g., HR, respiratory rate, overall ventilation, energy substrates, mood, and motivation; Noble and Robertson, 1996), have a decisive influence on the perceived exertion, which then, as an integrative predictor, determines exercise tolerance and fatigue during endurance exercise (Baron et al., 2008; Crewe et al., 2008, Marcora and Staiano, 2010). The fact that, so far, none of the numerous physiological, biochemical, and cognitive models used to explain peripheral and central fatigue can fully clarify the limitations and determinants of endurance performance confirms this assumption (Abbiss and Laursen, 2005; Weir et al., 2006; Enoka and Duchateau, 2008; Shephard, 2009). Here, Non-linear analysis of HRV may provide a different perspective on complex cardiovascular interaction during endurance performance.

In addition, the results of the present study, and the assumptions described above, indicate that the RPE could also be seen as a part of this feedback loop (Fontes et al., 2015; Pageaux, 2016). Indeed, RPE acts as a strong predictor of exercise tolerance and fatigue and substantially determines the duration until exercise termination with constant workload (Horstman et al., 1979; Eston et al., 2007; Crewe et al., 2008; Nakamura et al., 2008; Noakes, 2008; Marcora and Staiano, 2010). Thus, perception of effort should be considered not only as a marker of exercise intensity, but also as a factor limiting endurance performance (Pageaux and Lepers, 2016), for example as cardinal exercise stopper during high-intensity aerobic exercise rather than severe locomotor muscle fatigue or intolerably unpleasant muscle pain (Staiano et al., 2018). Furthermore, the RPE increases significantly with the onset of exercise and increases further linearly during prolonged exercise under HC until voluntary exhaustion. In the NC condition, the course could be better characterized as exponential. However, the behavior of the RPE increase corresponds to the results of other studies and shows a rather linear trend in the course of the prolonged exercise with its highest values at voluntary exhaustion (Eston et al., 2007; Crewe et al., 2008; Nakamura et al., 2008; Fontes et al., 2010; Pires et al., 2011). Hence, the termination of endurance exercise seems to be caused by a conscious decision-making process in which the perception of effort plays an important role (Staiano et al., 2018).

As a proxy of afferent feedback, the RPE, which increased in the time course of the exercise during both conditions and with an intensified increase during HC, reflects a copy of the resulting efferent motor command, which is processed in sensory brain areas (Tucker, 2009; Williamson, 2010; Pageaux, 2016). This approach implies that the regulatory mechanism compares context-specific and teleanticipatory feedforward estimations of the internal load situation with permanent psycho-physiologically afferent feedback of the peripheral and central subsystems and organ systems; this results in a perception of effort. Thus, the function of the brain and the subjective RPE as associated markers for the protection of organismic homeostasis are considered to be of crucial importance for exercise tolerance, endurance performance, and the control of performance output during endurance exercise (e.g., pacing strategies in closed-loop exercises) (St Clair Gibson et al., 2006; Abbiss and Laursen, 2008; Tucker, 2009; Tucker and Noakes, 2009). In this context, the Non-linear analysis of HRV could provide a new perspective for the integrated and holistic consideration (as promoted by leading experts in the field) of regulatory and fatigue processes during exercise (Smirmaul et al., 2010; Marino et al., 2011; Venhorst et al., 2018), and can also describe the influence of different environmental conditions such as normobaric hypoxia. Overall, the application of DFA may provide a new possibility to analyze the relationship between different modes of exercise, environmental conditions, and the altered cardiovascular (autonomic) regulation. This could be useful in response to the concern over strongly decreased variability and weak reproducibility of amplitude dependent time- and frequency-domain HRV measures during exercise (Persson and Wagner, 1996; Tulppo et al., 2005; Millar et al., 2009; Gronwald et al., 2019a).

## Limitations

The generalizability of the presented findings in this pilot study is limited due to the small number of participants. In addition, only male cyclists were included in order to exclude the confounding influence of sex. Although the DFA is a recognizably useful diagnostic method, especially because linear approaches of spectral analyses are unable to reveal changes in HRV that are related to the non-linear interaction of physiological mechanisms, their detailed physiological interpretation remains unclear (Silva et al., 2017). We are also aware that exercise physiology during exercise is too complex and too dependent on certain conditions to be broken down into a single key measure. Nevertheless, DFA analysis of HRV may represent a suitable approach with a qualitative view of physiologic exercise regulation, and it may be useful in combination with other applicable internal and external load measures for training diagnostics and monitoring (Gronwald et al., 2019a). In addition, DFA extracts intrinsic properties of HRV dynamics, where the total variability is disregarded. Although log–log plots of fluctuations vs. window size will be shifted up or down regarding total variability, the DFA-alpha1 (slope of the curve) will remain unaffected. Hence, the presented results can be assumed to be related to the cardiac autonomic modulation and not affected by different mean HRs (Peng et al., 1995; Sandercock and Brodie, 2006; Silva et al., 2017). Lastly, in order to minimize the environment’s external influence and to enable coupling processes in the presented study and previous studies (Gronwald et al., 2018, 2019a,b), respiration was not prescribed and controlled. Allowing spontaneous breathing might have been a limitation, but it was necessary as we aimed to analyze individual responses during exercise as stated by Blasco-Lafarga et al. (2017).

## Conclusion

The present data show for the first time that under both normoxic and normobaric HCs prolonged exercise until voluntary exhaustion provokes a lower total variability combined with a reduction in the amplitude and correlation properties of RR fluctuations, which may be attributed to increased organismic demands. These findings verify a demand-dependent change to anti-correlated or uncorrelated/stochastic behavior of the RR-intervals during prolonged exercise until voluntary exhaustion. This loss of complexity in the time series during exercise could be related to the disruption of the equilibrium and interaction between the two branches of the autonomic nervous system shown by DFA-alpha1. Additionally, normobaric hypoxia provoked higher demands and a more pronounced loss of correlation properties at an earlier stage during the exercise regime, implying an accelerated alteration of cardiac autonomic regulation. Hence, DFA analysis provides a complementary systemic view (Ahn et al., 2006) of cardiovascular regulation in the context of complex models of exercise and fatigue during different environmental conditions. From a practical point of view, the given approach may help to elucidate the influence of different training regimes under varied conditions and with athletes of different performance levels on complex autonomic regulation and therefore offer a new assessment and monitoring tool of the functional state of athletes (Gronwald et al., 2019a).

## Data Availability

The datasets for this study will not be made publicly available because only raw data of HRV (rr-intervals) exist.

## Ethics Statement

This study protocol was approved by the ethics committee of the University Clinic of Halle (Saale), Medical Faculty of Martin Luther University of Halle-Wittenberg and was performed in accordance with the guidelines of the Helsinki World Medical Association Declaration. Following explanation of any risks and benefits associated with the study, all participants approved the study and written informed consents were obtained.

## Author Contributions

TG and KH conceived and designed the study details. TG conducted and analyzed the data collection and wrote the first draft of the manuscript. OH and KH reviewed and edited the draft critically. All authors read and accepted the final version of the manuscript.

## Conflict of Interest Statement

The authors declare that the research was conducted in the absence of any commercial or financial relationships that could be construed as a potential conflict of interest. The reviewer SL declared a past collaboration with the authors TG, OH, and KH to the handling Editor.
